# Does low-intensity pulsed ultrasound accelerate phasic calcium phosphate ceramic-induced bone formation?

**DOI:** 10.1590/acbe380023

**Published:** 2023-02-20

**Authors:** Lanying Sun, Xiaoshuang Guo, Qibao Wang, Zhongshuai Shang, Yi Du, Guodong Song

**Affiliations:** 1 Jinan Stomatological Hospital – Oral Implantology Center – Jinan, China.; 2 Plastic Surgery Hospital – Oral and Maxillofacial Surgery Department – Chinese Academy of Medical Sciences & Peking Union Medical College – Beijing, China.; 3 Jinan Stomatological Hospital – Department of Endodontics – Jinan, China.

**Keywords:** Ultrasonic Waves, Osteogenesis, Biocompatible Materials

## Abstract

**Purpose:**

Low-intensity pulsed ultrasound (LIPUS) has been used to stimulate the healing of the fresh fracture, delayed union, and non-union in both animal and clinical studies. Besides, biphasic calcium phosphate ceramic (BCP) is a promising biomaterial for bone repair as it shows favorable biocompatibility, osteoinduction, and osteoconduction. However, scarcity is known about the combined effect of LIPUS and BCP on bone formation.

**Methods:**

The combined effect of LIPUS and BCP was studied in a beagle model. Twelve dogs were used. BCP granules without any additions were implanted into bilateral erector spinae muscles. One side is the BCP group, while the counterlateral side is LIPUS + BCP group. Histological and histomorphometric analyses, and quantitative real-time polymerase chain reaction were evaluated.

**Results:**

Compared with BCP alone, the LIPUS + BCP showed no advantages in early bone formation. Furthermore, the Notch signaling pathway-related mRNA has no significant difference between the two groups.

**Conclusions:**

The preliminary results showed that the BCP, which has intrinsic osteoinduction nature, was an effective and promising material. However, LIPUS has no enhanced effect in BCP induced ectopic bone formation. Furthermore, LIPUS has no effect on the Notch signaling pathway. Whether costly LIPUS could be used in combination with BCP should be a rethink.

## Introduction

Critical-sized bone defects and non-unions requiring bone grafting are still challenges in clinical routine, as they could prolong the length of hospitalization and increase health-care expenditure. Commonly, natural bone grafts and synthetic biomaterials are used in such a scenario. However, the use of natural bone grafts is limited by the intrinsic disadvantages of donor site damage, the limited amount of bone mass (autografts), immunogenicity, and disease transmission (xenografts)^1^. Therefore, the exploration of effective biomaterials is imperative for clinical use. Among the plethora of available biomaterials, biphasic calcium phosphate ceramics showed satisfactory efficacy and reached clinical use^2^. However, the efficiency and mechanical strength of biomaterials-induced bone formation are still far from satisfactory.

Except for bone graft, several auxiliary therapies have also been studied to accelerating bone regeneration. Among them, low-intensity pulsed ultrasound (LIPUS) has drawn increasing attention because of its non-invasive physical stimulation for therapeutic applications. Several animal studies^3^^-^ 6 proved positive effects of LIPUS for promoting faster healing of femoral fracture in aged or osteoporotic rats. A similar healing effect was also confirmed on sheep and rabbit models^7^^,^ 8.

The combined effect of LIPUS with ceramic biomaterials has also drawn attention in the recent one decade. Researchers studied whether LIPUS could accelerate ceramic biomaterials-induced bone formation. Wu *et al*.^9^ proved in vitro that LIPUS facilitated the ingrowth, proliferation, and early differentiation of MC3T3-E1 mouse preosteoblasts in silicon carbide porous ceramic scaffold. Iwai *et al*.^10^ applied LIPUS to porous hydroxyapatite ceramic implanted in the femoral condyle of rabbits and detected significantly increased osteoblast number and mineralized volume. However, whether LIPUS could facilitate BCP-induced bone formation is still unclear. Thus, we designed an experimental beagle model to evaluate the effectiveness of the combination of LIPUS and BCP as a valuable combinatorial tool for clinical use.

## Methods

### Materials

BCP ceramics granules (Φ 5 mm × 8 mm) were purchased from the National Engineering Research Center for Biomaterials (Chengdu, Sichuan, People’s Republic of China). 60wt.% hydroxyapatite and 40wt.% β-tricalcium phosphates were prepared using wet precipitation and hydrogen peroxide (H_2_O_2_) foaming method and then sintered for 3 hours at 1,100 °C. The macropore size was 380 ± 50 μm, overall porosity (%vol) was 70.8%, and specific weight was 0.970 g/cm^3^ (Table 1)^11^. The morphology was analyzed by scanning electron microscope (SEM, Philips XL20, Dutch).

**Table 1:** Relevant parameters of biphasic calcium phosphate.

Parameters	Ratio of hydroxyapatite/ β-tricalcium phosphates	Macropore size (μm)	Overal porosity (%)	Specific weight (g/cm^3^)
Biphasic calcium phosphate	6:4	380 ± 50	70.8	0.970

### Animal model

**Figure 1:**
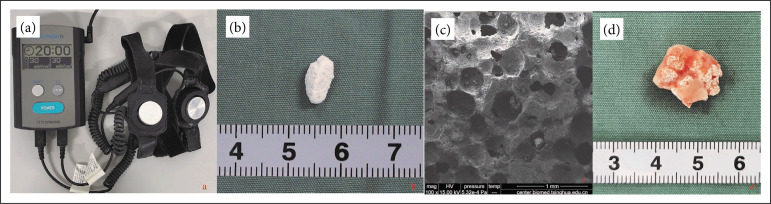
**(a)** Low-intensity pulsed ultrasound device; (**b and c**) the gross scanning electron microscopegraphs of the biphasic calcium phosphategranule; **(d)** the gross graph of the excavated specimen.

The experiment was conducted using a self-control method. Twelve beagle dogs (10 months old, 10-12 kg, half male and half female) were used. General anesthesia was achieved by abdominal injection sodium pentobarbital (30 mg/kg). The animals were placed in the prone position, and the back was shaved. After sterilizing, an 8-cm longitudinal incision was made in the dorsum, four intramuscular pockets were made, and eight BCP granules were inserted into one pocket. The wound was closed layer by layer (Fig. 1). Penicillin was intramuscularly injected consecutively during the first three days after surgery to prevent infections. The present study was approved by the Institutional Animal Care and Use Committee of our hospital. Animal experiments were carried out following the National Institutes of Health guide for the care and use of laboratory animals.

### Low-intensity pulsed ultrasound treatment

The LIPUS device (Osteoron IV, Japan) was daily used on the right side of the operated back. The apparatus was set at 1 KHz frequency, 0.3 W/cm^2^ intensity, duty cycle of 20% for 2 0min. After four, eight and 12 weeks, four dogs were sacrificed randomized by an overdose of sodium pentobarbital, and the implants were harvested (Fig. 1a).

### Histological evaluation of neo-ossification

Excavated samples were halved for both histological and quantitative real-time polymerase chain reaction (qRT-PCR) study. Half of the samples were fixed in 4% paraformaldehyde, and then decalcified using 10% ethylene diamine tetraacetic for one~two weeks, and after dehydrated in ascending concentrations of ethanol and embedded in paraffin. Tissue sections were sliced, and the cross section was stained with hematoxylin/eosin (HE) staining. Occurrence of bone or osteoid tissue formation was assessed by one laboratory technician and one experienced pathologist, who were unknown to the assignment of groups. Histomorphometry was performed according to previous report^11^^-^ 13. Incidence of neo-ossification (In) was defined as bone/osteoid filled pores in randomly selected non-overlapping fields at a magnification of 5× (Eq. 1).


P1 = the number of bone and osteoid filled pores / the number of overall pores (1)

Pores which were partially exhibited in one figure were not calculated.

Volume of neo-ossification (Vn) was defined as the percentage of bone/osteoid tissue inside the pores (P2) in 10× magnified fields (Eq. 2).


Vn = (bone and osteoid area within pore 1#/area of the pore 1# +bone and osteoid area within pore 2#/area of the pore 2# +...+ bone and osteoid area within pore n#/area of the pore n#)/n (2)

n: the number of intact pores in every 10× magnified field.

The area of the pores and the bone/osteoid area inside the pores were measured respectively using ImageJ software.

### Quantitative real-time polymerase chain reaction

After implantation for four and eight weeks, BCP granules were excavated and stored in RNAlater Stabilization Solution (Thermofisher, Waltham, United States of America). The central part of the sample was used for RNA isolation using the RNeasy kit (Qiagen, Venlo, Netherlands). Complementary DNA (cDNA) was obtained by reverse transcription of 1,000 ng of total RNA using the TransScript One-Step gDNA Removal and cDNA Synthesis SuperMix kit (Transgen, China). qRT-PCR was performed on a light cycler PCR machine (Roche, Switzerland) using SYBR green I master mix (Roche, Switzerland) three times. The following thermocycling conditions were used: initial denaturation at 95 °C for 10 minutes; followed by 40 amplification cycles (denaturation at 95 °C for 15 sec, annealing at 60 °C for 60 sec and extension at 72 °C for 45 sec).

As showed in Table 2, the primer sequences were referred to previous published articles or designed using the Primer 6 software (Hewlett-Packard, Madrid, Spain). The expression of osteogenesis and the Notch signaling pathway-related genes was normalized to GAPDH levels. The data were analyzed using the 2-AACq method (25) and LightCycler® 96 software version 1.1 (Roche Diagnostics GmbH). The experiment was performed in triplicate, and the results are expressed as relative fold changes.

**Table 2:** Primers for the quantitative real-time polymer chain reaction.

Gene	Forward primer	Reverse primer
Alkaline phosphatase	5’-GACATGCAGTACGAGCTGAACAGG-3’	5’-GTACCCGCCAAAGGTGAAGACG-3’
Osteocalcin	5’-GGTGGTGCAACCTTCGTGTC-3’	5’-GCATACTTCCCTCTTGGGCT -3’
Osteopontin	5’-ACGATTTCCAGTTCAGAGCAGT-3’	5’-GGGTAGAGTTCTCAGCGTCG-3’
Jagged1	5’- ACAATGGTGGCTCATGTCGT-3’	5’- TTTCGGGCTATGTTGCAGGT-3’
Hey1	5’-GCGGATGAGAATGGAAACTTG-3’	5’-AGTCCATAGCAAGGGCGTG-3’
RUNX2	5’-GATGCGTCTTCCCGTGGAC-3’	5’-CCCAGTTCTGAAGCACCTGA-3’
GAPDH	5’-TCCATCTTCCAGGAGCGAGA-3’	5’-TCCGATGCCTGCTTCACTAC-3’

### Statistics

Light micrograph images were analyzed using ImageJ software, and the result was reported as mean±standard deviation (SD). The statistical analysis was calculated using SPSS 20.0 software (SPSS, Inc., California, United States of America), and variation between samples was analyzed using paired-t test if the numbers are normally distributed. If not, the Wilcoxon signed-rank test was used. p < 0.05 was considered as significant difference.

## Results

### Gross and scanning electron microscope graphs showed structures of biphasic calcium phosphate granules

The BCP were porous with similar macropores and micropores. The surface of BCP exhibited roughness, and adjacent macropores was connected. The macroporosity and interconnectivity facilitate colonization of cells and perfusion of nutrition (Figs. 1b and 1c).

### Gross evaluation of explants

No surgical complications including infection/wound dehiscence were detected. Under compression of erector spinae muscle, the majority of implanted BCP granules were accumulated in a cylinder shape and surrounded by muscular and fibrous connective tissue (Fig. 1d). At four weeks, although connected, the BCP particles were intact and could be easily separated. At eight weeks, the BCP particles were incorporated with fibrous tissue and could be easily sliced down after one-week decalcification. After 12 weeks, the BCP particles were surrounded by thick fibrous capsule and need at least two-week decalcification before histologic analysis. The volume of samples remained unchanged, with no significant macroscopic increase or decrease.

### Histomorphometrical evaluation of neo-ossification

**Figure 2:**
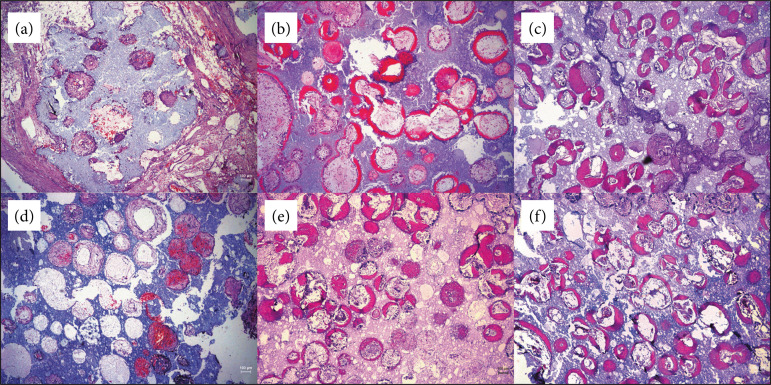
Hematoxylin/eosin staining of the biphasic calcium phosphate granules. (**a-c**) Biphasic calcium phosphate group; (**d-f**) biphasic calcium phosphate + low-intensity pulsed ultrasound group. The left column was four weeks, the middle column was eight weeks,while the right column was twelve weeks.

Figure 2 showed the overview of bone or osteoid tissue formation at four, eight and 12 weeks after implantation. Bone or osteoid tissue was scarcely observed at four weeks, only fibroblast-like, monocyte-like could be seen. At eight weeks, bone or osteoid tissue was stained in red as it was acidophilia. Newly formed bone or osteoid tissue was in the peripheral areas, while fibrous tissue and neovascularization dwelled in the central area. Furthermore, fibrous tissue and neovascularized tissue also existed in the connection between macropores.

**Figure 3:**
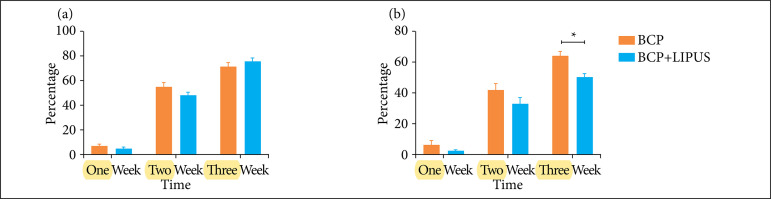
The histomorphometrical result of neo-ossification. **(a)** The incidence of bone or osteoid tissue. **(b)** The volume of the bone or osteoid tissue in macropores.

BCP: biphasic calcium phosphate; LIPUS: low-intensity pulsed ultrasound.

Figure 3a depicted the incidence of bone or osteoid tissue in BCP and BCP + LIPUS groups. At four and eight weeks, BCP group showed a slightly higher incidence of bone or osteoid tissue when compared with LIPUS additional group. However, the difference was not statistically significant.

Figure 3b illustrated the volume of bone or osteoid tissue in BCP and BCP + LIPUS groups. Quantification of % of BCP-induced ectopic bone formation in macropores showed that, at the fourth and eighth weeks, there was no difference in BCP and BCP + LIPUS groups. At the 12th week, the volume of neo-ossification in BCP group was higher than in the BCP + LIPUS group.

### Quantitative real-time polymerase chain reaction of osteogenesis-related genes

**Figure 4:**
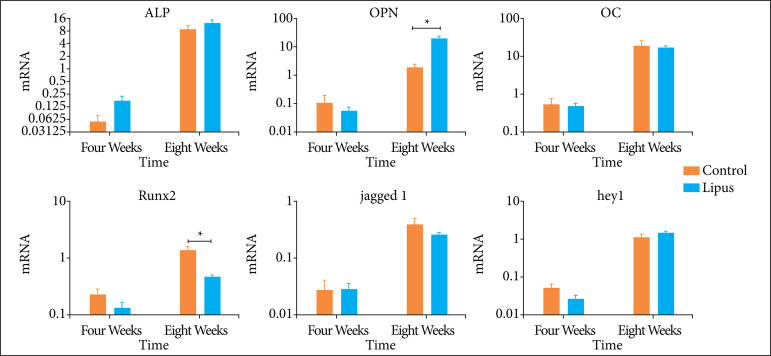
The expression of mRNA of osteogenesis and the Notch signaling pathway-related genes.

BCP: biphasic calcium phosphate; LIPUS: low-intensity pulsed ultrasound; ALP: alkaline phosphatase; OC: osteocalcin; OPN: osteopontin; RUNX2: Runt related transcription factor 2.

Alkaline phosphatase (ALP), osteocalcin (OC), and osteopontin (OPN) were selected as classic osteogenesis-related genes. At the fourth week, both BCP and BCP + LIPUS groups expressed the lowest level in all genes. At the eighth week, the mRNA expression in the targeted genes increased parallelly. However, for ALP and OC mRNA, no differences were detected between groups. The LIPUS might add positive effect in increasing the mRNA expression of OPN (Fig. 4).

### Quantitative real-time polymerase chain reaction of Notch signaling pathway-related genes

Guo *et al*.^11^ conducted a preliminary experiment and found the pivotal role of Notch signaling pathway in BCP-induced ectopic bone formation. Thus, in the present study, Notch signaling pathway-related genes were also evaluated. There were no differences in Jagged1/hey1 mRNA expression between BCP and BCP + LIPUS groups. Runt related transcription factor 2 (RUNX2) is a critical factor in the regulation of osteogenic differentiation. BCP + LIPUS showed decreased expression of RUNX2 mRNA (Fig. 4).

## Discussion

LIPUS is a therapeutical ultrasound which can be transmitted as acoustical pressure waves in tissues. This low-intensity ultrasound is non-thermogenic, without destruction in biological tissue^14^. In 1994 and 2010, United States of America and United Kingdom separately approved the clinical use of LIPUS for fresh fracture healing and non-unions. From then on, because miscellaneous experimental studies supported the positive effect of LIPUS in accelerating bone healing^15^, cartilage repair^16^^,^ 17, and even in neuron growth^18^ and erectile dysfunction^19^, LIPUS has become a highly recommended device with an increasing scope of clinical application.

In clinical trials, several studies proved that LIPUS enhanced healing rate among fresh fractures and nonunion > one year^15^^,^ 20 ^,^ 21. Moreover, compared with the whole population, older patients (≥ 60) with fracture risk factors treated with LIPUS had similar heal rates^22^. However, more and more clinical investigations are contentious with contradictory results. Hannemann *et al*.^23^ concluded that, although LIPUS accelerated bone healing when considering the time to radiological and clinical union, current evidence from randomized clinical trials was inadequate to support the benefit of LIPUS in reducing the incidence of nonunions for acute fracture treatment. Lou *et al*.^21^ conducted a meta-analysis regarding the effect of LIPUS on distraction osteogenesis and found no beneficial effect of LIPUS in reducing treatment time or complication rate. When it comes to non-unions, Leighton *et al*.^24^ summarized that LIPUS could benefit patients for whom surgery is high risk due to no spontaneous healing of definite fracture non-unions was expected. Thus, LIPUS is recommended alternative to surgery for this scenario.

Increasingly updated studies question the traditional findings. Busse *et al*.^25^ conducted a multi-center RCT across North American academic trauma centers and concluded that LIPUS after tibial fracture fixation did not accelerate neither radiographic healing nor functional recovery. Furthermore, a clinical practice guideline published in 2017 issued a strong recommendation against LIPUS in fresh fracture or osteotomy and even non-unions.

In the present study, the gross observation and histomorphometrical calculation of ectopic bone formation showed no beneficial effect of LIPUS on BCP-induced ectopic bone formation. Furthermore, osteogenesis-related genes ALP/OC showed no difference between LIPUS + BCP and BCP groups. Although the mRNA expression of OPN was increased in the LIPUS + BCP group, the change was trivial. The synthesized results were insufficient to conclude a benefit of the combination of LIPUS and BCP as a combinatorial tool for clinical use. The finding is different from previous experiment by Fan *et al*.^26^. They studied the combined effect of LIPUS and porous BaTiO_3_/pTi scaffold and found that the adhesion, proliferation, gene expression of bone marrow derived stem cell were increased by LIPUS in vitro, and osteogenesis/osseointegration was promoted in large segmental defect repair of rabbit model.

The inefficiency of LIPUS in BCP-induced ectopic bone formation and the disparity of present and previous studies may be due to the difference of biomaterial and animal model. First, the similar mechanism may explain why LIPUS showed no adjuvant effect to BCP-induced bone formation. In the present study, BCP was used as porous scaffold. The mechanism of BCP and LIPUS-induced bone regeneration is overlapped. Several proposed theories of BCP-induced de new osteogenesis are:

- Ca^2+^ and PO_4_^3-^ ions released from BCP intrigue bone formation;
- Several growth factors including BMPs (Bone Morphogenetic Protein)/VEGF (Vascular Endotelial Growth Factor) absorbed by the porous ceramic promote bone formation and neovascularization;
- Surface structure physically stimulates cellular signaling pathway and thereby intrigues osteogenesis^27^^,^ 28.

Similarly, the mechanism of LIPUS-induced bone regeneration is also associated with ions uptake and angiogenesis. Tassinary *et al*.^29^ proved that LIPUS was capable of stimulating differentiation and mineralization of MC3T3-E1 cells through Ca^2+^ and PO_4_^3-^ uptake. Meanwhile, LIPUS can increase the mRNA level of vascular endothelial growth factor A^26^. Secondly, the ectopic bone formation model did not put the roles LIPUS exerted on the residual bone defect to good use. In the present study, ectopic bone formation rather than in-situ bone repair model was used. In in-situ repair model, biomaterials exert osteoinductive and osteoconductive function, while merely exerting osteoinductive effect in ectopic bone formation^30^. The biomechanical waves of LIPUS on residual bone defect can produce additional stimulation and promote bone regeneration. Thus, it may put both the biomaterial-induced bone integration/bone regeneration and residual bone-induced bone regeneration to good use. Thirdly, LIPUS does not always showed an additional effect to implantable materials. Similarly, Ishihara *et al*.^31^ studied the combined effect of LIPUS with statin in nasal bone defect and found not statistical difference between combined group from LIPUS alone or statin alone.

Notch is an important signaling pathway in bone repair and regeneration. Guo *et al*.^11^ reported that Notch signaling pathway also regulated in BCP-induced ectopic bone formation. In the present study, Notch signaling pathway related genes, Jagged1 and Hey 1 showed no change in the LIPUS + BCP group. The mRNA of RUNX2 even reduced in the LIPUS + BCP group. RUNX2 is also a pivotal downstream transcription factor which could be influenced by several signaling pathways, including Notch signaling pathway, BMP signaling pathway, Wnt signaling pathway. Contrary with previous study, BCP + LIPUS showed decreased expression of RUNX2 mRNA, which indicated the negative effect of LIPUS in BCP-induced ectopic bone formation.

The present experiment has several limitations. First, only ectopic bone formation was used. Further study of in-situ bone defect model is warranted to study the combinatorial effect of LIPUS and BCP. Second, the sample size was relatively small. Third, only one apparatus and one kind of BCP was studied on the present study.

## Conclusions

BCP, which has intrinsic osteoinduction nature, was an effective and promising material. However, LIPUS has no enhanced effect in BCP-induced ectopic bone formation. Furthermore, LIPUS has no effect with the Notch signaling pathway. Whether costly LIPUS could be used in combination with BCP should be rethink.
